# Supplementary material for: Scaling down the microbial loop: data‐driven modelling of growth interactions in a diatom–bacterium co‐culture
Source: Environ Microbiol Rep. 2021 Sep 19;13(6):945–54. doi: 10.1111/1758-2229.13010 (PMC9293018; doi:10.1111/1758-2229.13010)
Supplement: Supplementary file 1 — Fig. S1 Chlorophyll a content in Phaeodactylum tricornutum (Phtri) grown in co‐culture with the bacterium Pseudoalteromonas haloplanktis TAC125 and in the control. Co‐culture and P. tricornutum control were cultured in SS medium, with no carbon source addition. Error bars, standard deviation of three biological replicates. Fig. S2. Growth curves of the diatom P. tricornutum (Phtri) in spent bacterial medium (grey, 50% diluted bacterial spent medium, green 100% bacterial spent medium, violet, control grown in fresh SS medium). Error bars, SEM (standard error of the mean) of duplicate cultures. Different letters describe significant difference (ANOVA, post‐test: Tukey's multi‐comparative test, p < 0.05). Fig. S3. A. Growth curves of the bacterium P. haloplanktis TAC125 (PhTAC125) in 50% diluted and 100% of spent diatom medium. P. haloplanktis TAC125 negative control grown in SS medium (in red). Error bars, standard deviation of triplicate cultures. Different letters describe significant differences (ANOVA, post‐test: Tukey's multi‐comparative test, p < 0.05). B. Growth curves of the bacterium PhTAC125 grown in a medium containing diatom‐autoclaved biomass. Error bars, standard deviation of triplicate cultures. The asterisk indicates significant difference (t‐test, p < 0.05). Fig. S4. The effect of different initial amounts of DOM on bacterial growth, from the concentration of the original simulation ‘Original’ to five times this concentration ‘5×’. Table S1. Cell counts of P. haloplanktis TAC125 in the co‐culture experiments. Bacterium positive control, grown in SS + l‐glutamic acid, as the only carbon source; bacterium negative control, grown in SS with no additional carbon source and bacterium in co‐culture with the diatom P. tricornutum, with no carbon addition. Mean and standard error of three biological replicates. Table S2. List of model parameters used in the model. [file EMI4-13-945-s001.docx]

**Supplementary Material:** Scaling down the microbial loop: data-driven modelling of growth interactions in a diatom-bacterium co-culture

Giulia Daly^1^, Elena Perrin^2^, Carlo Viti^1^, Marco Fondi^2, 3 *^, Alessandra Adessi^1^.

^1^ Department of Agriculture, Food, Environment and Forestry, University of Florence, Piazzale delle Cascine 18, Florence, Italy

^2^ Department of Biology, University of Florence, Via Madonna del Piano 6, Sesto F.no, Florence, Italy

^3^ Centro Interdipartimentale per lo Studio delle Dinamiche Complesse, University of Florence, Italy

* Corresponding author

**Figure S1­­:** Chlorophyll *a* content in *Phaeodactylum tricornutum* (*Phtri*) grown in co-culture with the bacterium *Pseudoalteromonas haloplanktis* TAC125 and in the control. Co-culture and *P. tricornutum* control were cultured in SS medium, with no carbon source addition. Error bars, standard deviation of three biological replicates.

**Figure S2:** Growth curves of the diatom *P. tricornutum* (*Phtri*) in spent bacterial medium (grey, 50% diluted bacterial spent medium, green 100% bacterial spent medium, violet, control grown in fresh SS medium). Error bars, SEM (Standard Error of the Mean) of duplicate cultures. Different letters describe significant difference (ANOVA, Post-test: Tukey’s multi comparative test, p<0.05).

**Figure S3: A)** Growth curves of the bacterium *P.haloplanktis* TAC125 (PhTAC125) in 50% diluted and 100% of spent diatom medium. *P. haloplanktis* TAC125 negative control grown in SS medium (in red). Error bars, standard deviation of triplicate cultures. Different letters describe significant differences (ANOVA, Post-test: Tukey’s multi comparative test, p<0.05). **B)** Growth curves of the bacterium PhTAC125 grown in a medium containing diatom-autoclaved biomass. Error bars, standard deviation of triplicate cultures. The asterisk indicates significant difference (t-test, p < 0.05).

**Figure S4.** The effect of different initial amounts of DOM on bacterial growth, from the concentration of the original simulation “Original” to five times this concentration “5X”.

|  |  | |
| --- | --- | --- |
|  |  |  |
|  |  |  |
|  |  |  |
|  |  |  |
|  |  |  |

**Table S1**: Cell counts of *P. haloplanktis* TAC125 in the co-culture experiments. Bacterium positive control, grown in SS + L-glutamic acid, as the only carbon source; bacterium negative control, grown in SS with no additional carbon source and bacterium in co-culture with the diatom *P. tricornutum*, with no carbon addition*.* Mean and standard error of three biological replicates. n.a. =not available due to uncountable colonies in all tested dilutions.

| **Time (days)** | **Cell counts: CFU/ml** | | | | | |
| --- | --- | --- | --- | --- | --- | --- |
|  | **Mean** | | | **Standard error** | | |
|  | Bacterium positive control | Bacterium negative control | Bacterium in  co-culture | Bacterium positive control | Bacterium negative control | Bacterium in  co-culture |
| 0 | 1.26 × 10^5^ | 1.59 x 10^5^ | 3.79 x 10^5^ | 5.45 × 10^4^ | 4.50 x 10^4^ | 2.39 x 10^5^ |
| 7 | n.a. | 2 x 10^5^ | 3.06 x 10^5^ | n.a. | 3.26 x 10^4^ | 7.31 x 10^4^ |
| 14 | 6.7 × 10^7^ | 3.92 x 10^5^ | 4.85 x 10^5^ | 1.82 × 10^7^ | 6.24 x 10^4^ | 2.03 x 10^5^ |
| 21 | 8.28 × 10^6^ | 5.11 x 10^5^ | 2.67 x 10^6^ | 1.13 × 10^6^ | 9.39 x 10^3^ | 5.84 x 10^5^ |
| 28 | 2.92 × 10^6^ | 1.47 x 10^5^ | 2.67 x 10^6^ | 6.97 × 10^5^ | 2. 81 x 10^4^ | 4.30 x 10^5^ |

**Table S2:** List of model parameters used in the model.

| **Parameter** | **Description** | **Fitted values** | **Unit** |
| --- | --- | --- | --- |
| $\upsilon_{\mu}^{D}$ | Maximal diatom growth rate | 0.098995093331007 | d^-1^ |
| ${CC}^{D}$ | Diatom carrying capacity | 16.689447443410732 | cells mL^-1^ |
| $\upsilon_{\delta}^{D}$ | Maximal diatom death rate | 0.001984229462814 | d^-1^ |
| $\upsilon_{\mu}^{B}$ | Maximal bacterial growth rate | 0.179068667164805 | d^-1^ |
| ${CC}^{B}$ | Bacterial carrying capacity | 7.397598065440601 | cells mL^-1^ |
| $K_{{DOM}_{E}}$ | Monod-like coefficient for DOM_E_ | 10.830696228140674 | cells mL^-1^ |
| $\upsilon_{\delta}^{B}$ | Maximal bacterial death rate | 0.017063187186277 | d^-1^ |
| $\lambda$ | DOM_E_ release rate | 0.044376089881716 | d^-1^ |
| $K_{{DOM}_{B}}$ | Monod-like coefficient for DOM_B_ | 0.239977111430301 | cells mL^-1^ |
| $\delta_{{DOM}_{B}}$ | DOM_B_ death rate | 0.004668152516864 | d^-1^ |
| $\delta_{{DOM}_{E}}$ | DOM_E_ death rate | 5.865508920503153 | d^-1^ |
| $\delta_{D}$ | Diatom death rate | 0.0019 | d^-1^ |
| $\delta_{B}$ | Bacterial death rate | 0.0170 | d^-1^ |
